# Ultrathin graphene oxide membranes on freestanding carbon nanotube supports for enhanced selective permeation in organic solvents

**DOI:** 10.1038/s41598-018-19795-z

**Published:** 2018-01-31

**Authors:** Seon Joon Kim, Dae Woo Kim, Kyeong Min Cho, Kyoung Min Kang, Junghoon Choi, Daeok Kim, Hee-Tae Jung

**Affiliations:** 1 Department of Chemical and Biomolecular Engineering (BK-21 Plus) & KAIST Institute for Nanocentury, Korea Advanced Institute of Science and Technology (KAIST), Daejeon, 34141 Korea; 2 Graduate School of EEWS, Korea Advanced Institute of Science and Technology (KAIST), Daejeon, 34141 Korea

## Abstract

Among the various factors required for membranes in organic solvent separations, the stability of membrane supports is critical in the preparation of membranes with universal chemical stability, mechanical flexibility, and high flux. In this study, nanoporous freestanding carbon nanotube (CNT) films were fabricated and utilized as supports for enhanced permeation in organic solvents. The excellent chemical stability of the CNT support allowed it to withstand various organic solvents such as toluene, acetone, and dimethylformamide. In addition, the structural stability and high pore density of CNT supports allowed the deposition of an ultrathin selective layer for an enhanced-flux membrane. Membrane performance was demonstrated by depositing a thin graphene oxide (GO) layer on the CNT support; GO was selected because of its high chemical stability. CNT-supported GO membranes effectively blocked molecules with molecular weight larger than ~800 g mol^−1^ while allowing the fast permeation of small molecules such as naphthalene (permeation was 50 times faster than that through thick GO membranes) and maintaining selective permeation in harsh solvents even after 72 hours of operation. We believe that the developed CNT support can provide fundamental insights in utilizing selective materials toward organic solvent membranes.

## Introduction

Membrane technology is expected to be highly effective at reducing the separation costs in various applications because of its lower power consumption and highly integrated facilities in comparison with other separations such as distillation and adsorption^1–6^. Utilizing the technology in chemically harsh conditions such as organic solvent systems is especially important, because the majority of impurities and products that need to be filtered in petroleum, chemical, and pharmaceutical industries are handled in harsh chemical conditions^7,8^. An important requirement for membranes to function properly in practical applications is retention of long-term stability along with high selectivity and high flux, as well as negligible swelling or degradation in harsh chemical conditions during operation.

To keep pace with the development of selective barrier materials for membranes, porous support materials that are stable in harsh chemical solvents must also be designed because the overall performance of a membrane is determined in large part by the properties of the support^9,10^. Support layers require appropriate pore structures for high flux and sufficient mechanical strength to support the ultrathin selective layer. Moreover, the support must be able to be mass-produced to reduce membrane costs. Polymeric supports such as nylon, polyacrylonitrile, polyimide, poly(ether ether ketone), and polyvinylidene fluoride are widely used materials in the industry^11,12^ because they can be fabricated on a large scale *via* a solution process such as phase inversion technique and can be rolled in a module because of their high flexibility. Ceramic supports such as alumina, silica, zirconia, and clay show excellent chemical stability in harsh organic and acidic solvents and can be packed in a hollow-fiber structure^13,14^. However, membrane supports of both categories need to overcome critical limitations: polymeric supports typically degrade and swell in harsh solvents^15^ and stability-reinforced structures tend to fail when applied to long-term operation, whereas ceramic supports are typically brittle and have high manufacturing costs^15^. Polymeric supports are also difficult to use in processes requiring high temperatures. As the thickness of selective layers becomes ultrathin to maximize the permeation rate, such as in the case for carbon sieves^16^, zeolite^17^, metal organic frameworks^18^, and layered two-dimensional materials^1,19–23^, small failures of porous supports can significantly degrade the separation performance of the selective layer and that of the overall membrane. Therefore, the development of a novel porous support with high chemical and mechanical stability, high porosity, and flexibility is required, as well as manufacturability *via* practical methods. In this manner, carbon materials are a promising candidate as robust supports for organic solvent membranes since carbon films are highly stable in chemicals and can be fabricated on a large scale into flexible modules without breaking, therefore overcoming the intrinsic limitations of polymeric and ceramic supports.

Herein, we demonstrated that a freestanding entangled carbon nanotube (CNT) film can be an effective porous support in organic solvent separation. A freestanding CNT support with a highly nanoporous and fibrous network with intrinsic nanopores (30 nm diameter size) was fabricated by simply filtering aqueous CNT dispersions using a vacuum-filtration technique. The CNT supports displayed outstanding chemical stability, as well as high resistance toward various organic solvents and acidic and basic solutions without any sign of swelling. Furthermore, the high flexibility allowed the supports to be rolled into cylinders. Graphene oxide (GO) showed synergetic property in the formation of selective layers on the CNT supports where GO layers of ~40 nm thickness were deposited *via* sequential vacuum filtration. Small molecules such as naphthalene displayed a high rate of permeation through the CNT-supported GO membranes (GO-CNT membranes), while larger molecules such as brilliant blue were nearly blocked; this result implies selective permeation. Because a very thin GO membrane using the CNT support can be applied, permeation rates were much higher compared with those in previous reports on GO used in organic solvent separation^19^. GO-CNT membranes also showed high chemical stability, retaining their performance in harsh organic solvents over prolonged exposure times. While the porous structure and high mechanical strength of CNT ‘buckypaper’ films (elastic modulus: ~2 GPa^24^) have been widely utilized for water filtration membranes^25^, this study provides fundamental insight for the utilization of their excellent chemical stability to organic solvent membranes.

## Results

### Membrane fabrication

**Figure 1:**
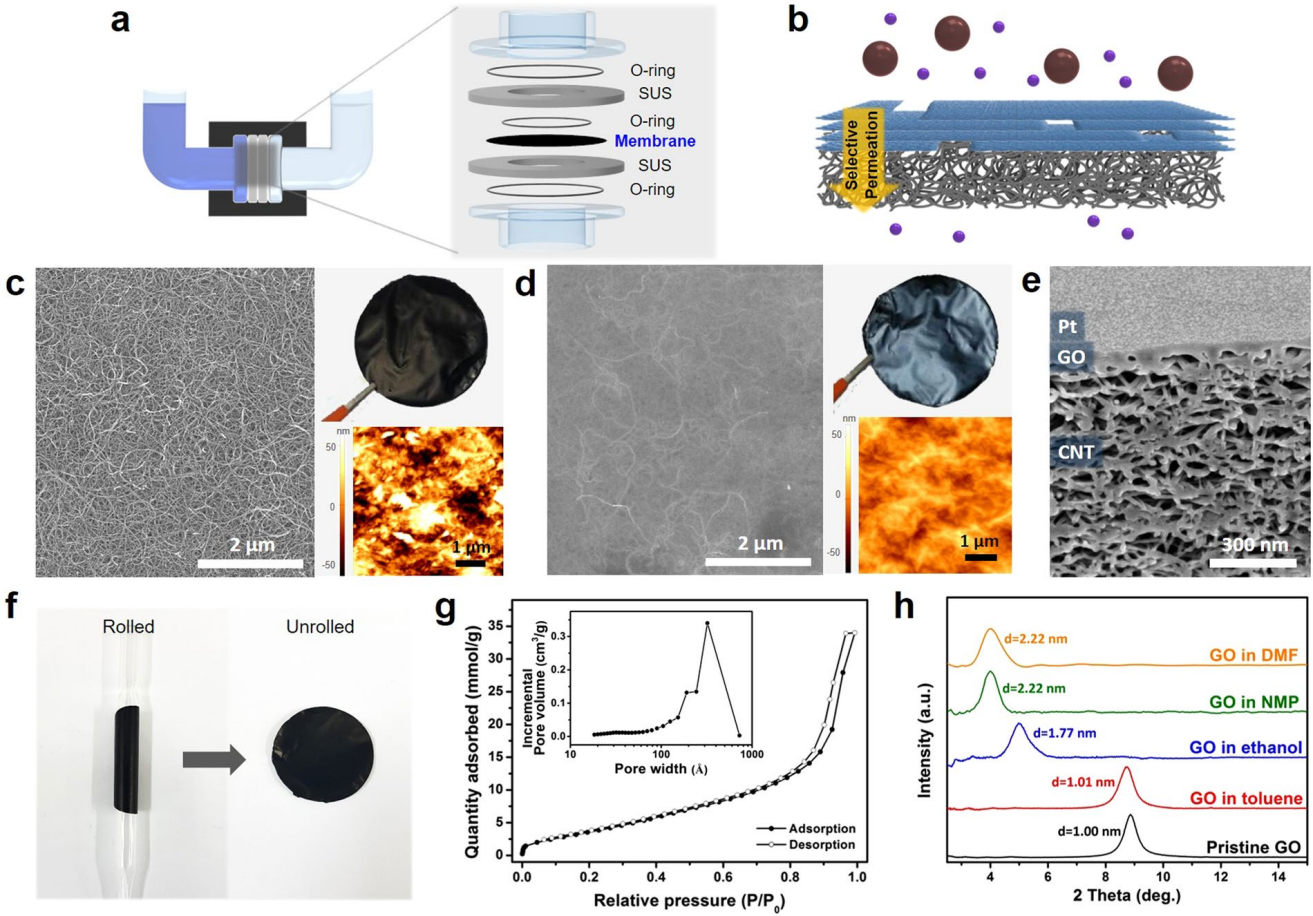
Testing apparatus and structural characterization of the GO-CNT membrane. (**a**) Schematic of the permeation apparatus and its components. (**b**) Schematic of a GO-CNT membrane for selective permeation. (**c**,**d**) Top-view SEM image of the (**c**) CNT membrane and (**d**) GO-CNT membrane. Top-right insets show the optical image of a freestanding membrane with a diameter of 35 mm, and the bottom-right insets show the surface roughness measured by AFM. (**e**) Magnified cross-sectional SEM image of a GO-CNT membrane coated with a Pt layer. (**f**) Images of a CNT support rolled into a cylinder with a radius of 3.5 mm and then unrolled back into its original form. (**g**) Ar isotherm of the CNT support at 87 K, the inset of which shows the pore size distribution. (**h**) XRD patterns of pristine GO (black), and GO immersed in various organic solvents: toluene (red), ethanol (blue), NMP (green), and DMF (orange).

In this work, concentration-gradient-driven permeation was employed rather than other techniques to focus on the fundamental aspects and chemical stability of the GO-CNT membranes. To investigate the diffusion rate, we used equipment with a U-shaped chamber as shown in Fig. 1a and Supplementary Fig. S1. The equipment is composed of stainless-steel plates with O-rings to perfectly seal the membrane, and it was carefully designed to withstand harsh solvents. GO thin films were utilized as the selective layer to demonstrate the performance of the CNT support in this study (Fig. 1b). The performance is due to the high chemical stability of GO and its ease of deposition as an ultrathin film on the CNT support *via* sequential vacuum filtration.

Initially, a CNT support was fabricated by vacuum filtration to form a smooth and uniform film, in which the prepared CNT support film displayed a black texture with a film diameter of 35 mm. The SEM image (Fig. 1c) shows that the CNT support film was composed of well-dispersed entangled nanotubes, with an average diameter of individual nanotubes of about 20 nm (See Supplementary Fig. S2). Porous spaces were also clearly observed between individual nanotubes. The roughness of typical CNT support films was equivalent to a root-mean-square (RMS) value of 30.0 nm as measured by atomic force microscopy (AFM); the roughness was due to the several tens of nanometer-scale spaces between the nanotubes. Since CNT was not oxidized in any way during film preparation, XPS measurements typically revealed a sharp C1s peak at 284.8 eV without other peaks from oxidized carbons, indicating that carbon atoms of CNT are dominantly sp^2^ bonded (See Supplementary Fig. S3). As discussed earlier, a thin GO layer was deposited on the CNT film to produce a GO-CNT membrane, and it is important to note that GO was selected because of its high chemical stability against organic solvents; other types of thin membranes can also be employed provided they have good chemical stability. The deposition of GO film on the CNT support is easily observable from the color change of the films, depending on the extent of GO deposition; the GO-CNT membrane had a shiny black texture, while the CNT support film was matte and black (see Fig. 1d). The SEM image (Fig. 1d) shows that the entire surface of the CNT support was uniformly covered with a GO layer. Non-selective defects such as pinholes and cracks were not observed on the surface of the GO-CNT membrane. While the GO films were deposited on the entire surface of the CNT support, a few strands of CNTs could be observed through the GO layer because of the extreme thinness of the GO film (Fig. 1d). Successful deposition of a uniform GO layer on top of the CNT support was also verified by AFM measurements. The RMS value of the GO-CNT membrane was 10.2 nm, which is much smaller than that of a pristine CNT membrane (RMS of 30.0 nm), indicating the deposition of a well-stacked GO film on the nanoporous CNT support. A smooth height profile at the GO layer boundary indicates conformal GO deposition on the CNT surface (See Supplementary Fig. S4).

Focused ion beam (FIB) was used to reveal and observe the cross-sectional stacking morphology of the GO-CNT membrane (Fig. 1e). A Pt layer was coated on the surface of the GO-CNT membrane prior to FIB treatment to ensure film integrity and to enhance the contrast between adjacent layers. Two distinct layers were observed in the cross section. The bulk of the membrane was composed of a mesoporous CNT structure with 12 μm thickness (See Supplementary Fig. S5), and the GO film was conformally stacked above the CNT layer. As the cross-sectional image does not clearly reveal the thickness of the GO layer due to similar contrast, AFM measurements were conducted for precise measurement, revealing a GO layer thickness of 40 nm (See Supplementary Fig. S6). The GO-CNT membranes were also characterized by Raman spectroscopy using a 514 nm excitation laser (See Supplementary Fig. S7), which revealed combined peaks of CNT^26^ and GO^27^ at 1347 cm^−1^ (D), 1581 cm^−1^ (G), and 2682 cm^−1^ (2D). Compared with the Raman peaks pristine CNT, those of GO-CNT have a larger full width at half-maximum, as well as higher intensity ratios I_D_/I_2D_ and I_G_/I_2D_, because peaks from GO overlap with the peaks of CNT.

The flexibility of the CNT supports was demonstrated by rolling the fabricated CNT films into cylinders with a radius of 3.5 mm (Fig. 1f). The CNT films easily returned to their original form after unrolling, and we could repeat the cycle several times without producing any cracks or defects on the CNT support. Since film-type membranes need to be integrated into rolled modules (such as spirally wound or hollow-fiber configurations) for industrial applications^28,29^, the high flexibility and good mechanical properties of the CNT support film may be very helpful in its practical utilization. Since the porosity of the membrane support is one of the key parameters that govern the permeation through the entire membrane, Ar gas adsorption isotherms were obtained to investigate the inner pore structure of the CNT support (see Fig. 1g). The CNT support displayed a type V isotherm, with a large uptake (up to 35 mmol g^−1^) at high relative pressures (P/P_0_ > 0.9) and a small degree of hysteresis, which corresponds to a surface area of 228 m^2^ g^−1^. The pore size distribution (inset of Fig. 1g) indicates that mesopores of 30 nm size and originating from the void spaces between individual nanotubes were dominant.

Because selective permeation of molecules through the multilayer GO film is determined by the interlayer spacing in various solvents^19^, XPS and XRD spectra for GO were investigated. Since individual graphitic layers in CNTs are known to not swell in all types of solvents, XRD measurements for a pristine CNT support were not performed. XPS measurements of GO (See Supplementary Fig. S8) revealed two dominant peaks at 284.8 eV (C–C) and 286.8 eV (C–O), with smaller peaks at 288.4 eV and 289.3 eV corresponding to oxygen functionalities (C=O and –COOH, respectively)^30^; these indicate the hydrophilic nature of GO. Prior to the XRD measurements, each membrane was immersed in the solvents for at least 24 hours to ensure full intercalation in the GO interlayers. Pristine GO produced a single peak at 2θ = 8.8°, whereas the peak of GO membranes immersed in the organic solvents toluene, ethanol, *N*-methylpyrrolidone (NMP), and dimethylformamide (DMF) appeared at 2θ values of 8.7°, 5.0°, 4.0°, and 4.0°, respectively (Fig. 1h). Each peak corresponds to an interlayer spacing of 1.00, 1.01, 1.77, 2.22, 2.22 nm for pristine GO, GO immersed in toluene, ethanol, NMP, and DMF, respectively. Because of the hydrophilic nature of GO, solvents with large polarity easily intercalated between layers, resulting in an interlayer spacing larger than that of pristine GO interlayers. In contrast, solvents with small polarity such as toluene barely intercalated between GO interlayers, which thus displayed interlayer spacing (~1 nm) similar to that of pristine GO^30^. Considering the van der Waals radius of the carbon and oxygen atoms in solid GO layers, the cutoff diameter of GO-CNT membranes in polar solvents may be in the range of 1.3–1.7 nm, depending on the degree of swelling.

### Chemical stability of membranes

**Figure 2:**
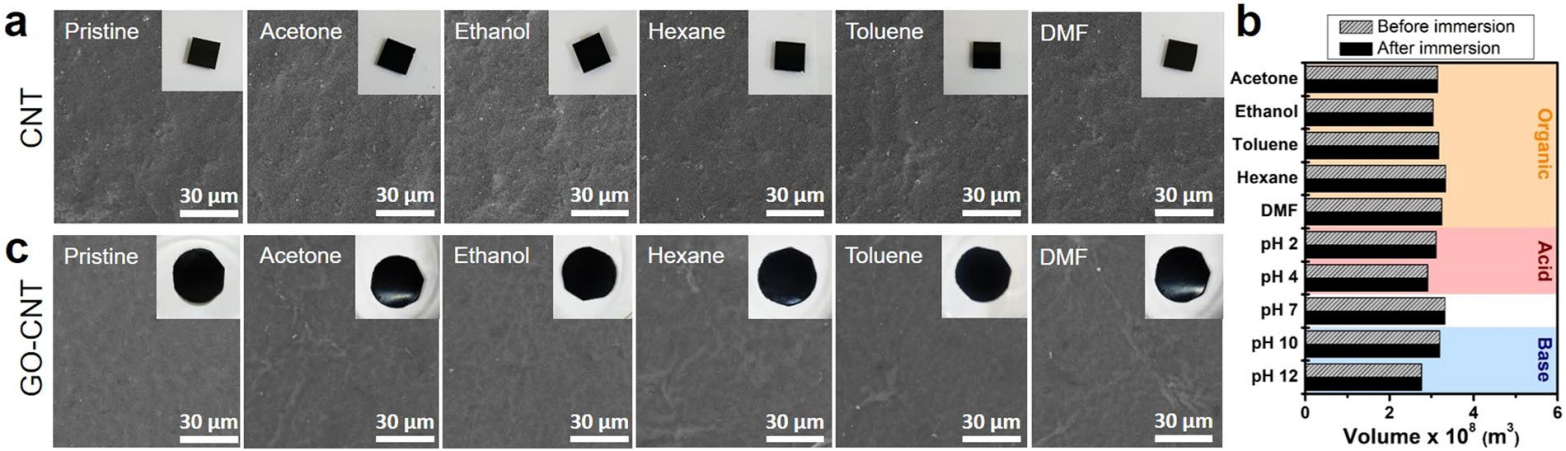
Stability of CNT supports and GO-CNT membranes in various types of solvents. (**a**) Top-view SEM images of the surfaces of pristine CNT supports and those after immersion in various solvents for 24 h. Insets show optical images of the supports after immersion. (**b**) Measurements of the geometric volume of CNT supports before (gray, pattern) and after immersion (black, solid) in solvents (organic, acidic, and basic) for 24 h. (**c**) Top-view SEM images of the surfaces of pristine GO-CNT membranes and those after immersion in various solvents for 24 h. Insets show optical images of the supports after immersion.

Although the chemical stability of membranes is highly important, fabrication of supports with swelling resistance to universal solvents as well as long-term stability remains challenging. To demonstrate the high stability of our CNT supports, they were immersed for extended periods in various organic solvents (acetone, ethanol, toluene, hexane, and DMF) and in acidic and basic solutions widely used to dissolve chemicals. The surface of the pristine CNT supports and those after 24 hours of immersion in various solvents are shown by the SEM images in Fig. 2a. No sign of defects or cracks were apparent and the original chemical properties of pristine CNT supports were retained as shown by FTIR and Raman spectra (Supplementary Fig. S9). Furthermore, the CNT supports did not show any visible swelling or degradation after immersion in organic solvents (insets of Fig. 2a). To quantitatively measure the resistance of CNT supports toward swelling in solvents, the geometric volume of individual supports was compared (shown in Fig. 2b). CNT support films were cut into rectangles with approximate dimensions of 0.5 cm × 0.5 cm, and the area and thickness of each film was measured prior to immersion. The films were immersed in the organic solvents and in acidic, neutral, and basic aqueous solutions (pH 2, 4, 7, 10, and 12) for 24 hours, after which the volume was measured upon drying. The volumes of the CNT films in all solvents remained the same, indicating that the films did not swell in the solvents. These results demonstrate that the CNT supports have universal stability in various harsh conditions. The high stability of CNT supports may be attributed to the pi–pi stacking between CNT walls and the highly entangled structure of CNTs due to the nanometer-scale width and micrometer-scale length (See Supplementary Fig. S2). Furthermore, the stability of GO-CNT membranes were also demonstrated by immersion in various organic solvents. The surface of pristine GO-CNT membranes and those after 24 hours of immersion did not show any noticeable defects and visible signs of degradation after immersion (Fig. 2c). Also, aside from the nanoscale swelling of GO layers in polar solvents observed by XRD, macroscale swelling after immersion was not observed, and the chemical properties of GO-CNT membranes were well retained as shown by the Raman spectra in Supplementary Fig. S10. The results above demonstrate the outstanding swelling resistance and chemical stability of GO-CNT membranes in universal solvents.

### Permeation of solutes in organic solvents

**Figure 3:**
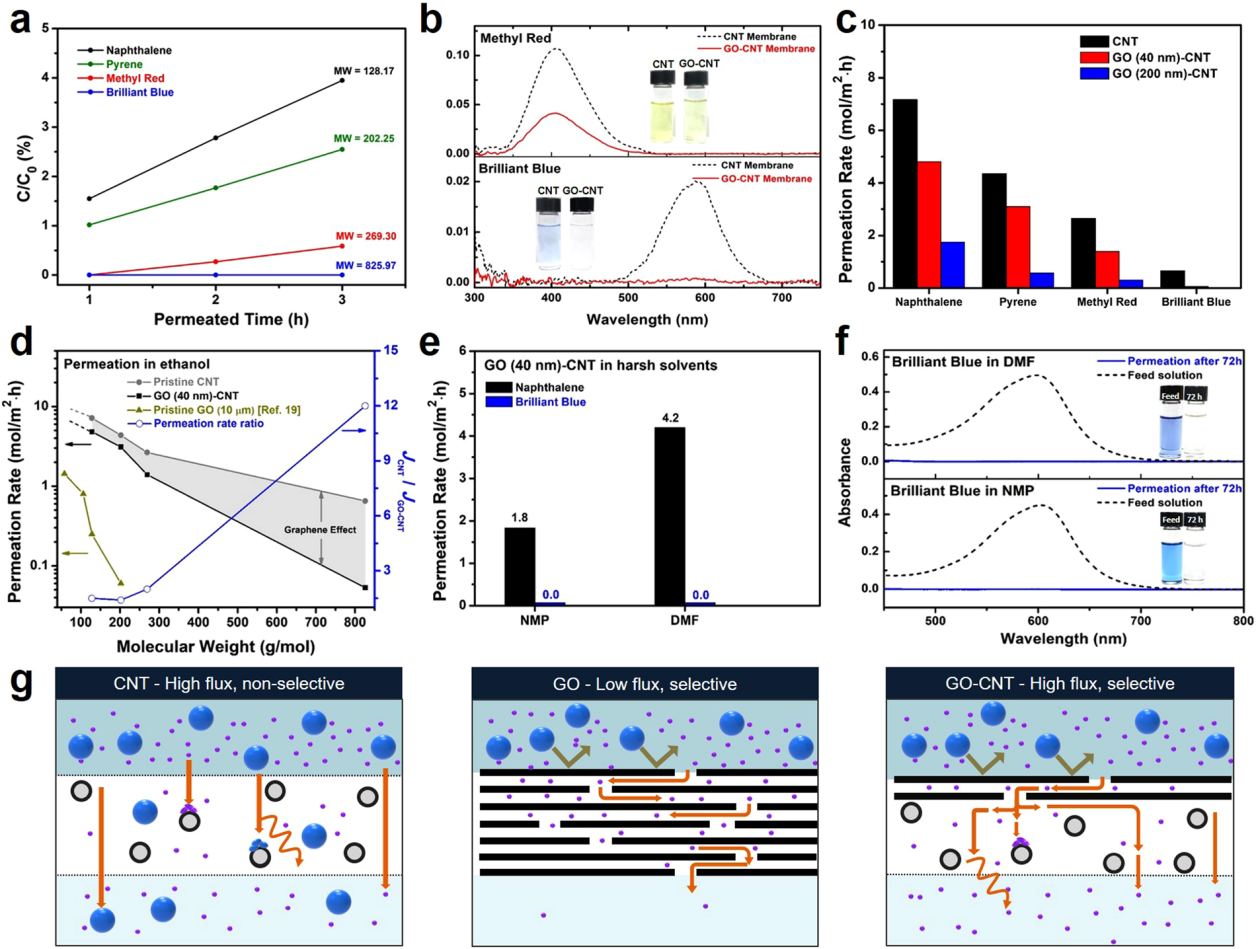
Performance of CNT support and GO-CNT membranes in organic solvents. (**a**) Permeation through a GO-CNT membrane represented as a function of solute concentration *vs*. permeation time. C_0_ is the feed concentration, C is the permeate concentration, and MW is the molecular weight. (**b**) UV–vis spectra of MR (top), and BB (bottom) permeating through CNT (black dashed line) and GO (40 nm)-CNT (red solid line) membranes in 24 h. Insets show actual images of permeating solutions. (**c**) Rates of permeation of various solutes in ethanol through CNT (black), GO (40 nm)-CNT (red), and GO (200 nm)-CNT (blue) membranes. Rates were normalized to those of 1 M feed solution concentration. (**d**) Permeation rates as a function of molecular weight. The blue line with hollow dots represents the ratio of rates of permeation through CNT to those of permeation through GO (40 nm)-CNT membranes. (**e**) Performance of GO-CNT membranes in NMP and DMF. Insets show actual images of permeated solutions. (**f**) UV–vis spectra of the feed solution (blue) and of BB through a GO (40 nm)-CNT membrane after 72 h (black) in NMP (bottom) and DMF (top). (**g**) Illustrated diffusion mechanism through a CNT support, thick GO membrane, and GO-CNT membrane.

To evaluate the separation performance of GO-CNT films in concentration-gradient-driven permeation in an organic solvent, solutes with various molecular weights and sizes were used to quantitatively study the diffusion phenomena through the GO-CNT membrane (See Supplementary Fig. S11): naphthalene (128.17 g mol^−1^), pyrene (202.25 g mol^−1^), methyl red (MR; 269.30 g mol^−1^), and brilliant blue (BB; 825.97 g mol^−1^). Neutrally charged solutes with molecular sizes smaller than the GO interlayer spacing were selected to minimize the influence of electric charge on permeation^31^. Figure 3a plots the relative concentration of each permeated solute in ethanol with respect to the feed concentration through a GO (40 nm)-CNT membrane *versus* diffusion time. Minimum amounts of aliquots for each permeated solution were collected at 1-hour time intervals to minimize its influence on the concentration gradient, and the concentration was measured by UV–vis absorption spectroscopy. While the permeate concentration linearly increased according to time regardless of the solute, the diffusion rate markedly varied depending on the size of molecules. For naphthalene, the smallest molecule in this study, the permeate concentration was 3.95% of the feed solution after 3 hours; whereas for MR, the permeated concentration was only 0.59% of the feed solution during the same period. To confirm whether the separation performance of GO-CNT membranes is effectively maintained during long term permeation, the selective diffusion of molecules through the GO-CNT membrane was further examined by comparing the UV–vis spectra of MR and BB through CNT (black dashed line) and GO-CNT (red solid line) membranes after 24 hours of permeation (Fig. 3b). For MR, significant permeation was observed through both CNT and GO-CNT membranes, with permeation rates of 20.8% and 8.8%, respectively. On the other hand, the permeation of BB largely decreased from 4.2% of CNT membrane to 0.3% after the addition of a GO layer even after 24 hours of permeation, indicating that GO effectively acts as an active layer for selective diffusion (See Supplementary Fig. S12). Immersion tests revealed that solute adsorption on CNT and GO-CNT membranes had a negligible influence in concentration change (See Supplementary Fig. S13), implying that concentration change was governed by membrane diffusion in both membranes.

To investigate the role of the GO layer and how the thickness of GO governs the separation performance of the membrane, the rates of molecular diffusion through pristine CNT supports, GO (40 nm)-CNT membranes, and GO (200 nm)-CNT membranes were measured (Fig. 3c). For comparison in overall, permeation rates in various concentrations were normalized to a fixed feed concentration of 1 M for all solutions (ethanol, NMP, and DMF), as the diffusion rate of molecules was linearly influenced by the feed concentrations (See Supplementary Fig. S14). The permeation rates through a CNT support were 7.2 (naphthalene), 4.4 (pyrene), 2.7 (MR), and 0.65 (BB) mol m^−2^ h^−1^, and all molecules tended to rapidly pass through without noticeable selectivity. The slight difference in permeation rates in CNT supports is expected to occur mainly because of size-dependent diffusion and adsorption on individual nanotubes^32^. With the addition of 40 nm GO, the permeation rates through a GO (40 nm)-CNT membrane were 4.8 (naphthalene), 3.1 (pyrene), 1.4 (MR), and 0.053 mol m^−2^ h^−1^ (BB). Small molecules still showed a high permeation rate, whereas diffusion of larger molecules such as BB became significantly hindered, leading to a very low permeation rate. In particular, we observed that increasing the thickness of the GO laminate to 200 nm greatly decreased the overall permeation rates, which were 1.8 (naphthalene), 0.57 (pyrene), 0.30 (MR), and 0.023 (BB) mol m^−2^ h^−1^. These results indicate that the thickness of GO laminates greatly influence the permeation rate and that an ultrathin GO layer is critical to fast, selective diffusion. Meanwhile, the thickness of the CNT layers influenced the total diffusion rate of the permeants where membranes with a 24 μm thick CNT layer showed a lower permeation rate compared to a 12 μm thick CNT layer as shown in Supplementary Fig. S15. The separation performance of each membrane was summarized in terms of the molecular weight of solutes filtered during concentration-gradient-driven permeation in Fig. 3d. When comparing the diffusion rate (*J*) through CNT and that through GO-CNT, The GO-CNT membrane with 40 nm GO thickness exhibited low selectivity for solutes with a molecular weight smaller than 300 g mol^−1^ (*J*_CNT_/*J*_GO-CNT_ < 2), as well as high selectivity to those with a molecular weight greater than 800 g mol^−1^ (BB; *J*_CNT_/*J*_GO-CNT_ = 12). From these results, we can deduce that the GO-CNT membrane acts as a molecular sieve with a molecular weight cutoff (MWCO) in the range of 300–800 g mol^−1^, effectively blocking solutes beyond this range. The outstanding performance of our GO-CNT membrane can be clearly seen by comparison with existing reports on GO membranes for organic solvent separation, which employ very thick GO films (10 μm). Because an ultrathin (40 nm) GO layer with the CNT support was possible, the GO-CNT membrane displayed a 16 times higher permeation rate for naphthalene and 50 times higher permeation rate for pyrene in ethanol while retaining the stability of the thick GO membranes^19^.

Permeation tests of GO-CNT membranes were also conducted in harsh solvents to investigate their dynamic stability during separation processes. The permeations of naphthalene and BB were compared to examine the selective permeation of the membranes. DMF and NMP, which are representative harsh solvents, were separately used to dissolve both solutes. Rates of naphthalene permeation through a GO (40 nm)-CNT membrane in NMP and DMF (Fig. 3e) were 1.8 mol m^−2^ h^−1^ and 4.2 mol m^−2^ h^−1^, respectively; the permeation of BB was undetectable in both solvents. The difference in the rates for naphthalene permeation in the two solvents seems be due to the variation in solvent–solute–membrane interactions. The permeation of BB was not detected with UV–vis spectra even after 72 hours of operation in both NMP and DMF (Fig. 3f), indicating that the GO-CNT membrane has long-term stability in harsh solvents. The mechanism of permeation through each membrane is illustrated in Fig. 3g. For the CNT support, permeation is governed by diffusion through interconnected nanopores and by fractional adsorption of solute molecules on the surfaces of CNTs. Since the interstitial pores (~30 nm size) are much larger than the solute molecules, selective permeation is negligible despite high permeation rates because of the short diffusion length. On the other hand, permeation through a GO membrane is governed by the diffusion of molecules through the interlayer spaces between each GO sheet, only allowing small molecules to pass through. Because the diffusion length is drastically increased with a small increase in thickness due to the large aspect ratio of the graphene sheets^20^, the permeation rate of molecules can be drastically reduced when the film thickness is increased. Permeation through a GO-CNT membrane combines the advantages of both materials. First, the ultrathin GO layer of 40 nm thickness exhibits selective permeation through molecular sieving. In this case, the overall diffusion length is minimal because of the thinness of the GO layer, allowing very fast permeation relative to that through thick GO membranes. Second, the nanopores of the CNT support ensures high permeation rates with short diffusion lengths along with structural integrity, functioning as an optimal support for an ultrathin selective layer.

### Permeation of mixed solutions

**Figure 4:**
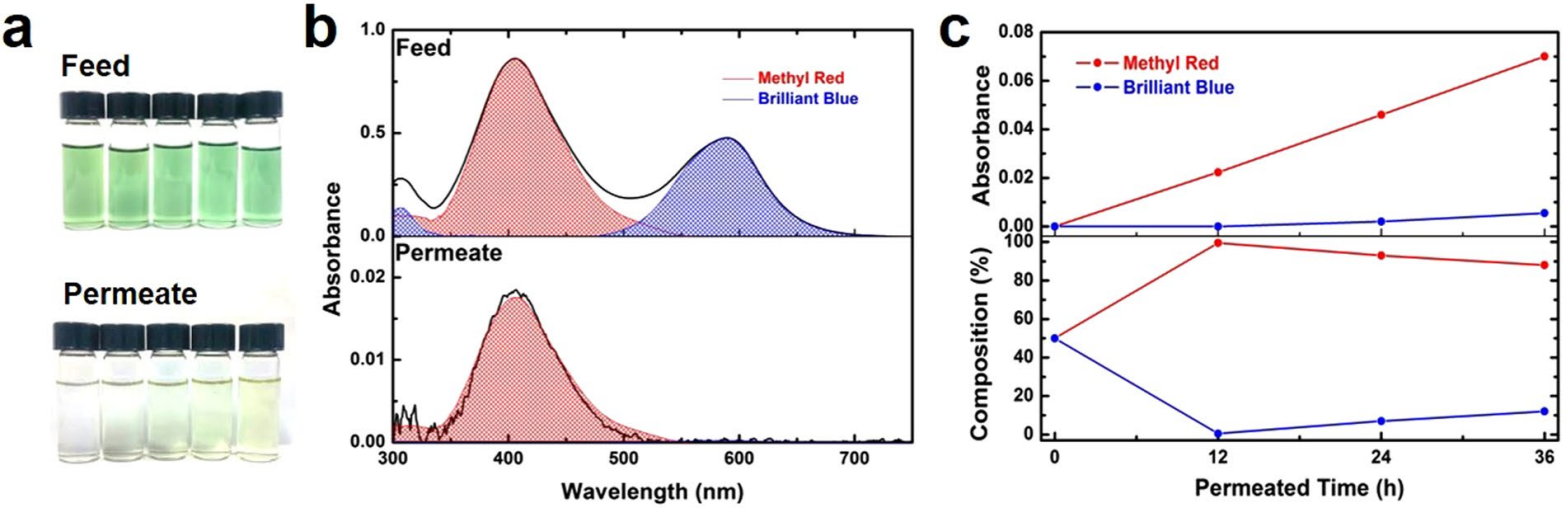
Separation of a multicomponent solution with GO-CNT membranes. (**a**) Pictures of the feed and permeate solutions at 12 h intervals from a feed solution of 50% MR and 50% BB. (**b**) UV–vis spectra of the feed solution and permeated solution after 12 h. (**c**) UV–vis absorbance and composition of the permeated solution at 12 h intervals.

To investigate whether selective permeation is achieved in the solutions, permeation tests were performed with a feed solution including two solutes, each smaller and larger than the pre-determined MWCO of approximately 800 g mol^−1^. This test determines whether the permeation behavior differs from those in tests in which only a single solute is used. A mixture of 50% MR and 50% BB in ethanol, which displayed a green color (Fig. 4a), was used as the feed solution. Rates of permeation through a GO (40 nm)-CNT membrane were measured. Permeation of MB dominated that of BB through the membrane; this could be visually seen as the gradual change in permeate solution color to yellow. UV–vis absorption measurements revealed that after 12 hours of permeation through the GO-CNT membrane (Fig. 4b), the composition of the permeate solution was about 99.5% MR and 0.5% BB. This indicates that separation of solutes can be achieved in a short time. Interestingly, the rate of MR molecule permeation through the GO-CNT membrane was found to be as fast (1.4 mol m^−2^ h^−1^) as that of permeation of single molecules in solution. Tests for extended periods (top graph, Fig. 4c) showed that the UV–vis absorbance of both MR and BB increases over time, with the slope of MR being much greater than the slope of BB because of the difference in permeation rates. The fraction of BB in the permeate solutions slowly increased at longer permeation times (Fig. 4c, bottom graph) due to the suppressed diffusion of BB through the GO membranes. These results indicate that GO-CNT membranes have a large potential application in practical separations using various solvents and mixtures. Furthermore, techniques for decreasing the GO interlayer, such as thermal treatment^19,33^, can be used to achieve even better separations.

## Discussion

In summary, we have developed a freestanding CNT support for organic solvent separations, which displayed high chemical stability while retaining high flux. The CNT supports did not display swelling behavior or degradation in various organic solvents, as well as acidic, or basic environments, showing outstanding stability. An ultrathin film of GO with 40 nm thickness was deposited on the CNT support, which was employed as a thin film composite membrane for organic solvent separation. GO-CNT membranes displayed enhanced permeation rates than GO paper in organic solvents for small molecules such as naphthalene, pyrene, and MR, which were comparable to those in pristine CNT membranes. Permeation rates were 50 times higher than measurements for thick GO membranes, demonstrating the high flux of the CNT support and the importance of an ultrathin selective layer. Simultaneously, the GO-CNT membranes exhibited selective permeation, efficiently blocking molecules larger than the interlayer distance, such as BB. The GO-CNT membranes also displayed selective permeation in harsh solvents such as NMP and DMF over long periods of operation because of the excellent stability of the CNT support. Selective permeation achieved in solutions also demonstrates the potential use of GO-CNT membranes in practical separation processes. Our results suggest that a composite membrane of CNT nanoporous supports combined with ultrathin selective layers can be an outstanding candidate for organic solvent membranes with high permeation rates, selective permeation, and high chemical stability.

## Methods

### Preparation of MWCNT dispersion and GO dispersion

The surfactant sodium dodecyl sulfate was dissolved in deionized water, and the mixture was stirred for 10 minutes to fully dissolve the powder. Multiwalled carbon nanotube (MWCNT) powders (Hanwha Chemical Co., Ltd.) were added to the surfactant solution to a concentration of 1 mg ml^−1^. The entire solution was then subjected to horn sonication for 1 hour to thoroughly disperse MWCNT. In order to remove the undispersed and agglomerated CNT powders, the obtained MWCNT aqueous solution was centrifuged at 8000 rpm for 10 minutes, and the supernatant was collected for use. The yield after centrifugation (CNT recovered in the supernatant) was about 20%. GO was fabricated from graphite powders (Graphit Kropfmühl GmbH, Germany) through the modified Hummer’s method, as described elsewhere. GO powders were collected and redispersed in deionized water to form a GO dispersion.

### Fabrication of CNT supports and GO-CNT membranes

GO-CNT membranes were prepared by vacuum filtration on an anodized aluminum oxide (AAO) support (Whatman, 200 nm pore size). Initially, the MWCNT dispersion was filtered to produce a CNT film, which was thoroughly rinsed with deionized water to remove residual surfactants. The CNT film was detached from the AAO supports to obtain a freestanding CNT support. To fabricate the GO-CNT membranes, a GO dispersion was poured on top of the CNT film before the CNT film was completely dried to deposit an ultrathin layer of GO by vacuum filtration. After the GO-CNT film was completely dried, it was detached from the AAO support to obtain a freestanding GO-CNT membrane.

### Measurement of permeation rates

Apparatus setup and the appropriate nomenclature (feed, permeate, etc.) for measuring the concentration-gradient-driven permeation through GO based membranes followed previous literatures^19,20^. The equipment is composed of a U-shaped glass chamber and stainless-steel plates embedded with O-rings to perfectly seal the membrane, which were carefully designed to withstand harsh solvents. Initially, CNT and GO-CNT membranes were clamped between stainless-steel plates and assembled with glass compartments into a U-shaped chamber, then one glass compartment was filled with the feed solution (200 ml) while the other compartment was filled with pure solvent (200 ml) at the same height to prevent external pressure-driven effects. Prior to each permeation test, each membrane was fully immersed in the solvent for at least 12 hours to completely wet the membrane. The concentrations of feed solutions in the main experiments were 1 M for naphthalene dissolved in ethanol, NMP, DMF and for pyrene dissolved in ethanol, while concentrations were 10 mg/L for methyl red dissolved in ethanol and for brilliant blue dissolved in ethanol, NMP, DMF. Solutions were constantly stirred using a magnetic bar to remove concentration gradients near the membrane surface. Small amounts of aliquots (3 ml) were taken at periodic intervals for analysis to minimize potential influence on the permeation tests, while equal amounts of both the feed solution and permeated solution were sampled to remove concentration gradient variation.

### Characterization

Cross sections of membrane samples were prepared using a focused ion beam (FIB, FEI Helios Nanolab 450 F1), and the surface images were obtained by scanning electron microscopy (SEM, FEI Nova320). Surface topology was analyzed using atomic force microscopy (AFM, Park Systems). Membrane film properties were also characterized using Raman spectroscopy (Horiba Jobin Yvon Aramis, 514 nm laser), X-ray photoelectron spectroscopy (XPS; Thermo Sigma Probe), and X-ray diffraction (XRD; SmartLab, Rigaku). Ar isotherm of CNTs film was measured using an ASAP 2020 physisorption analyzer (Micromeritics, USA) at 87 K. To analyze the pore-size distribution of CNTs film, DFT method was employed.

### Data availability

The data that support the plots within this paper and other findings of this study are available from the corresponding authors on request.

## Electronic supplementary material

Supplementary Information

## References

[1] Surwade SP (2015). Water desalination using nanoporous single-layer graphene. Nat. Nanotechnol..

[2] Degerman M, Jakobsson N, Nilsson B (2008). Designing robust preparative purification processes with high performance. Chem. Eng. Technol..

[3] Elimelech M, Phillip WA (2011). The future of seawater desalination: energy, technology, and the environment. Science.

[4] Das R, Ali ME, Hamid SBA, Ramakrishna S, Chowdhury ZZ (2014). Carbon nanotube membranes for water purification. Desalination.

[5] Nam YT, Choi J, Kang KM, Kim DW, Jung H–T (2016). Enhanced stability of laminated graphene oxide membranes for nanofiltration via interstitial amide bonding. ACS Appl. Mater. Interfaces.

[6] Kim DW, Choi J, Kim D, Jung H–T (2016). Enhanced water permeation based on nanoporous multilayer graphene membranes: the role of pore size and density. J. Mater. Chem. A.

[7] Darvishmanesh S (2011). Performance of solvent resistant nanofiltration membranes for purification of residual solvent in the pharmaceutical industry: experiments and simulation. Green Chem..

[8] Székely G, Bandarra J, Heggie W, Sellergren B, Ferreira FC (2011). Organic solvent nanofiltration: A platform for removal of genotoxins from active pharmaceutical ingredients. J. Membr. Sci..

[9] Ramon. GZ, Wong MCY, Hoek EMV (2012). Transport through composite membrane, part 1: Is there an optimal support membrane?. J. Membr. Sci..

[10] Jimenez-Solomon MF, Gorgojo P, Munoz-Ibanez M, Livingston AG (2013). Beneath the surface: Influence of supports on thin film composite membranes by interfacial polymerization for organic solvent nanofiltration. J. Membr. Sci..

[11] Marchetti P, Jimenez Solomon MF, Szekely G, Livingston AG (2014). Molecular separation with organic solvent nanofiltration: A critical review. Chem. Rev..

[12] Cacho-Bailo F, Seoane B, Téllez C, Coronas J (2014). ZIF-8 continuous membrane on porous polysulfone for hydrogen separation. J. Membr. Sci..

[13] Dong Z, Liu G, Liu S, Liu Z, Jin W (2014). High performance ceramic hollow fiber supported PDMS composite pervaporation membrane for bio-butanol recovery. J. Membr. Sci..

[14] Aba NFD, Chong JY, Wang B, Mattevi C, Li K (2015). Graphene oxide membranes on ceramic hollow fibers – Microstructural stability and nanofiltration performance. J. Membr. Sci..

[15] Van der Bruggen B, Mänttäri M, Nyström M (2008). Drawbacks of applying nanofiltration and how to avoid them: A review. Sep. Purif. Technol..

[16] Karan S, Samitsu S, Peng X, Kurashima K, Ichinose I (2012). Ultrafast viscous permeation of organic solvents through diamond-like carbon nanosheets. Science.

[17] Dong H (2015). High-flux reverse osmosis membranes incorporated with NaY zeolite nanoparticles for brackish water desalination. J. Membr. Sci..

[18] Sorribas S, Gorgojo P, Téllez C, Coronas J, Livingston AG (2013). High flux thin film nanocomposite membranes based on metal-organic frameworks for organic solvent nanofiltration. J. Am. Chem. Soc..

[19] Huang L, Li Y, Zhou Q, Yuan W, Shi G (2015). Graphene oxide membranes with tunable semipermeability in organic solvents. Adv. Mater..

[20] Joshi RK (2014). Precise and ultrafast molecular sieving through graphene oxide membranes. Science.

[21] Ren CE (2015). Charge- and size-selective ion sieving through Ti_3_C_2_T_x_ MXene membranes. J. Phys. Chem. Lett..

[22] Li W, Yang Y, Weber JK, Zhang G, Zhou R (2016). Tunable, strain-controlled nanoporous MoS_2_ filter for water desalination. ACS Nano.

[23] Akbari A (2016). Large-area graphene-based nanofiltration membranes by shear alignment of discotic nematic liquid crystals of graphene oxide. Nat. Commun..

[24] Zhang L, Zhang G, Liu C, Fan S (2012). High-density carbon nanotube buckypapers with superior transport and mechanical properties. Nano Lett..

[25] Sears K (2010). Recent developments in carbon nanotube membranes for water purification and gas separation. Materials.

[26] Zhang H–B, Lin G–D, Zhou Z–H, Dong X, Chen T (2002). Raman spectra of MWCNTs and MWCNT-based H_2_-adsorbing system. Carbon.

[27] Kim KH (2011). High quality reduced graphene oxide through repairing with multi-layered graphene ball nanostructures. Sci. Rep..

[28] Gabelman A, Hwang S–T (1999). Hollow fiber membrane contactors. J. Membr. Sci..

[29] Nicolaisen B (2002). Developments in membrane technology for water treatment. Desalination.

[30] Putz KW (2011). Evolution of order during vacuum-assisted self-assembly of graphene oxide paper and associated polymer nanocomposites. ACS Nano.

[31] Huang L (2016). Reduced graphene oxide membranes for ultrafast organic solvent nanofiltration. Adv. Mater..

[32] Kuo C–Y, Wu C–H, Wu J–Y (2008). Adsorption of direct dyes from aqueous solutions by carbon nanotubes: Determination of equilibrium, kinetics and thermodynamics parameters. J. Colloid Interface Sci..

[33] Jang J–H, Woo JY, Lee J, Han C–S (2016). Ambivalent effect of thermal reduction in mass rejection through graphene oxide membrane. Environ. Sci. Technol..

