# Supplementary material for: Ultrathin graphene oxide membranes on freestanding carbon nanotube supports for enhanced selective permeation in organic solvents
Source: Sci Rep. 2018 Jan 31;8:1959. doi: 10.1038/s41598-018-19795-z (PMC5792555; doi:10.1038/s41598-018-19795-z)
Supplement: Supplementary file 1 — Supplementary Information [file 41598_2018_19795_MOESM1_ESM.doc]

Supplementary Information for

Ultrathin graphene oxide membranes on freestanding carbon nanotube supports for enhanced selective permeation in organic solvents

Seon Joon Kim^1^, Dae Woo Kim*^, 1^, Kyeong Min Cho^1^, Kyoung Min Kang^1^, Junghoon Choi^1^, Daeok Kim^2^, Hee-Tae Jung*^, 1^

^1^Department of Chemical and Biomolecular Engineering (BK-21 Plus) & KAIST Institute for Nanocentury, Korea Advanced Institute of Science and Technology (KAIST), Daejeon 34141, Korea

^2^Graduate School of EEWS, Korea Advanced Institute of Science and Technology (KAIST), Daejeon, 34141 Korea

**Figures**


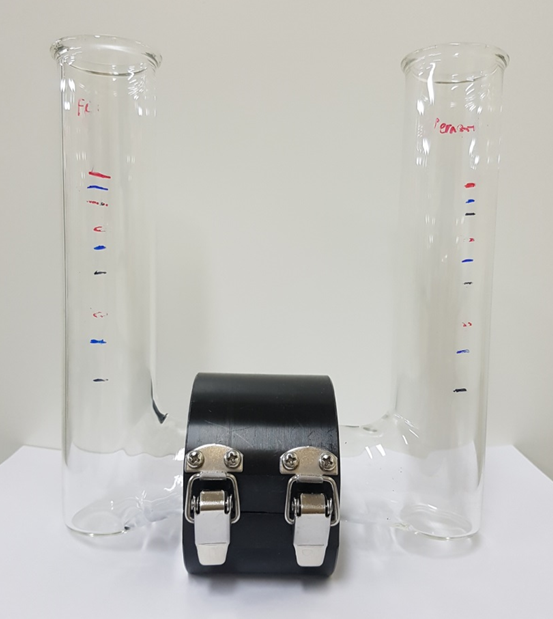


Supplementary Figure S1. U-shaped cylindrical apparatus for performing separation tests. Feed solutions were inserted on the left, while the permeated solution was collected from the right side.


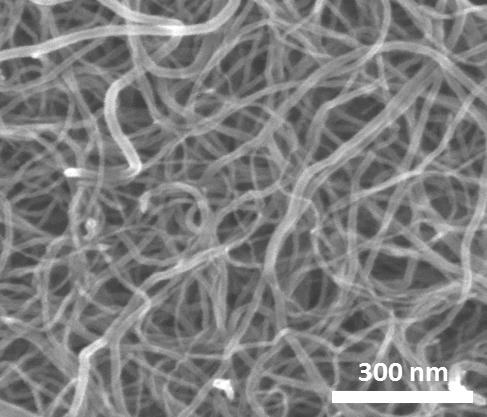


Supplementary Figure S2. High magnification image of a pristine CNT membrane. The diameter of each individual nanotube is about 20 nm.


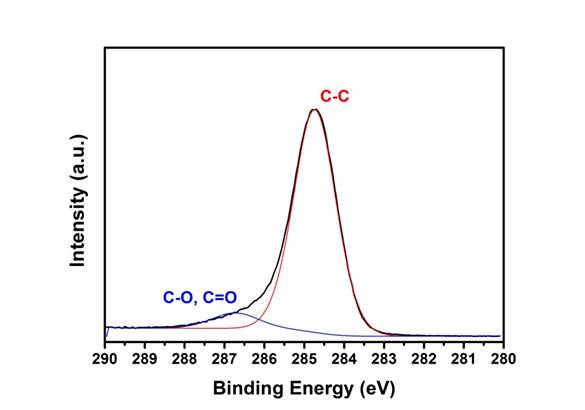


Supplementary Figure S3. XPS spectrum of the CNT support used in this study. C-C peaks are dominant with a small amount of oxygen functionalities on the surface.


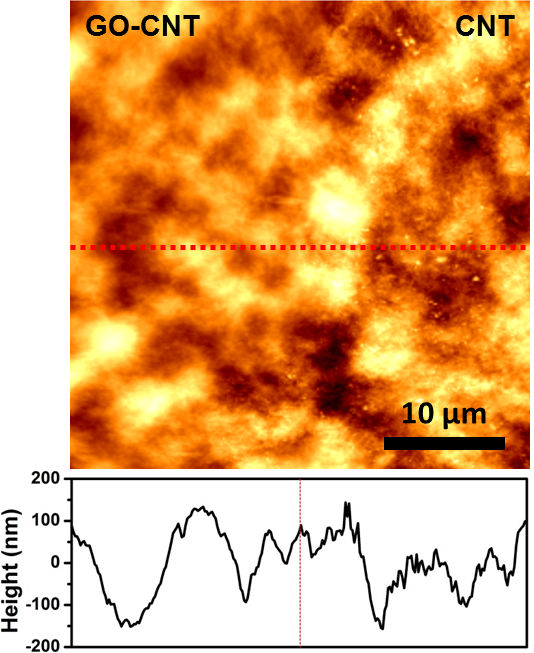


Supplementary Figure S4. Surface roughness at the GO boundary in a GO-CNT membrane. The right half represents a pristine CNT surface, and the left half represents CNT covered with GO. The region covered with GO displays a smoother surface compared to that of pristine CNT.


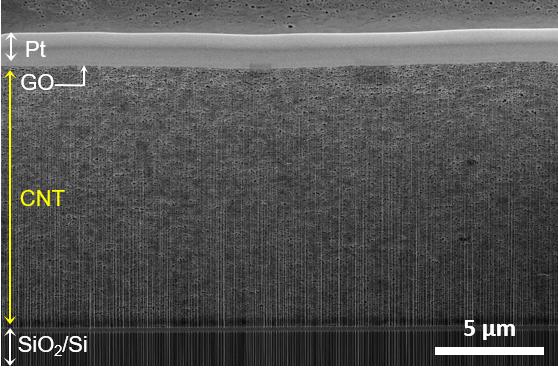


Supplementary Figure S5. Cross-sectional SEM image of a GO-CNT membrane coated with a Pt layer. GO-CNT membranes were placed on a SiO_2_/Si wafer substrate, then coated with Pt for clear observation and structural integrity.


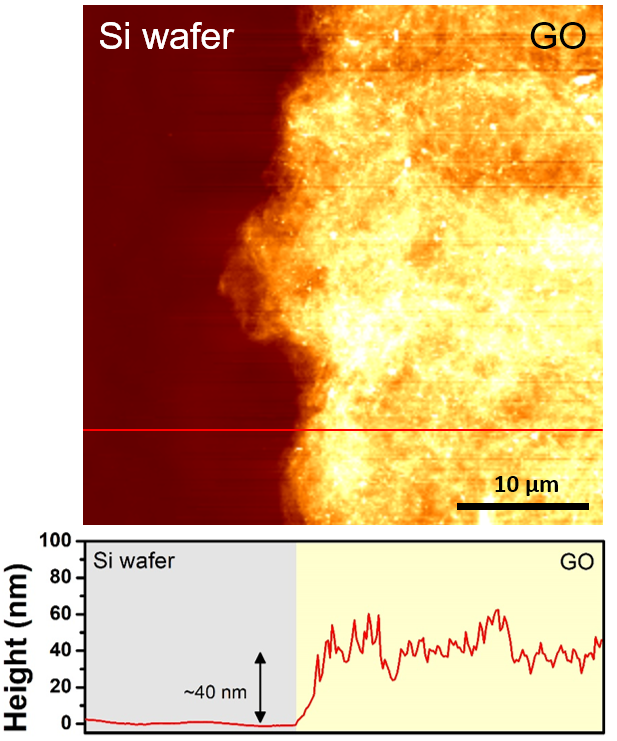


Supplementary Figure S6. Height profile of GO layer on a Si wafer. The GO layer of the GO-CNT membrane was separately coated on a Si wafer to reveal the thickness of ~40 nm.


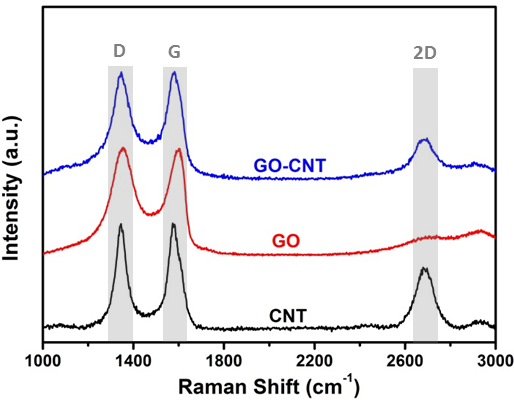


Supplementary Figure S7. Raman spectra of various components in the membrane. Representative D, G, and 2D peaks can be observed in GO-CNT (blue), GO (red), and CNT (black).


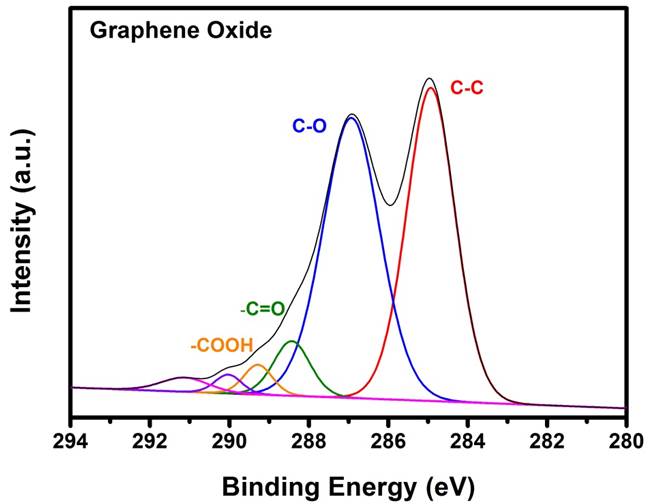


Supplementary Figure S8. XPS spectrum of the GO membrane used in this study. C-C bonds and C-O bonds are dominant, indicating a graphene basal plane highly decorated with oxygen functional groups on the surface.


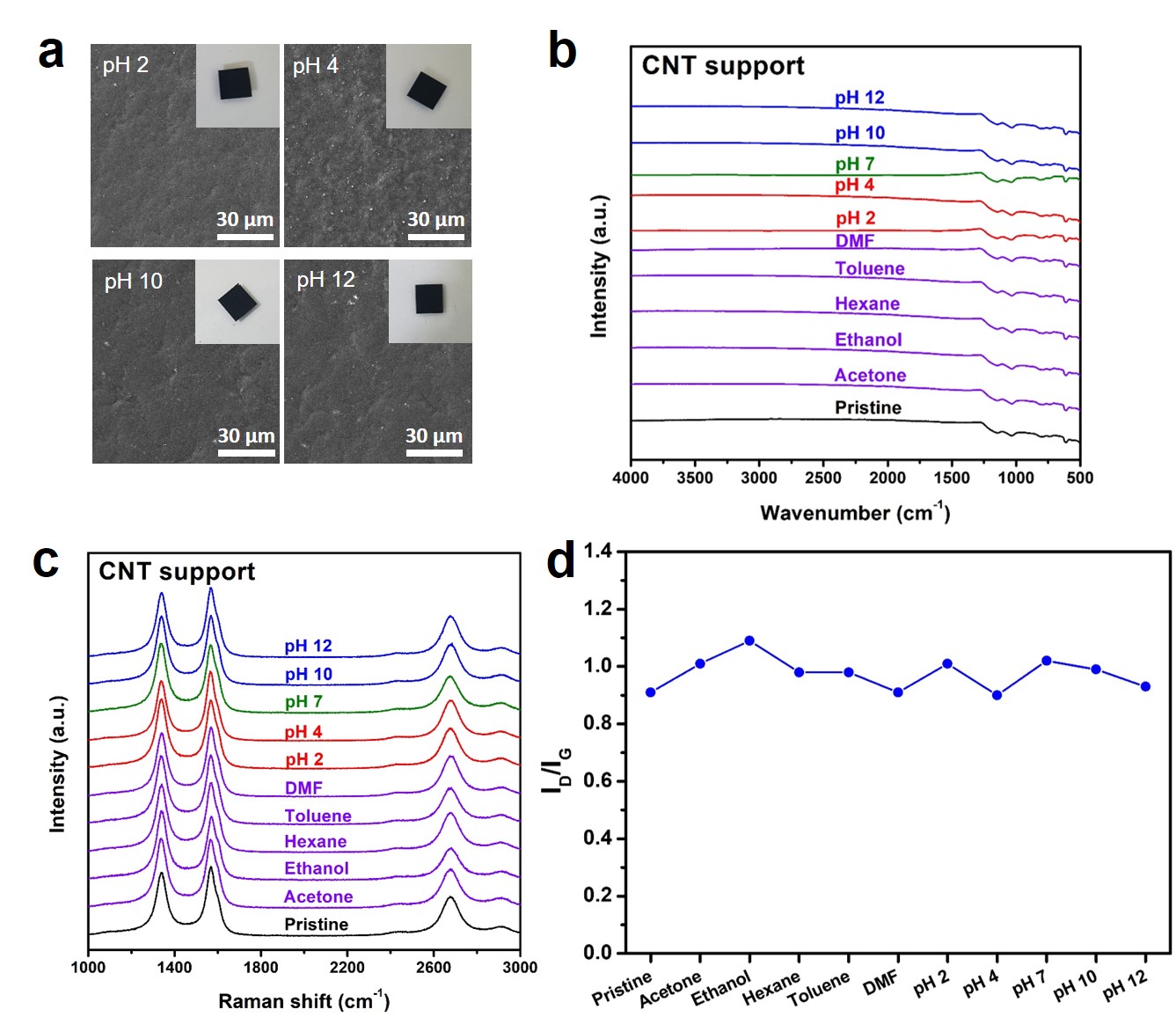


Supplementary Figure S9. Chemical stability of CNT supports. (a) Top-view SEM images of the surfaces of pristine CNT supports and those after immersion in acidic and basic solutions for 24 h. Insets show optical images of the supports after immersion. (b, c) (b) FTIR spectra, and (c) Raman spectra of pristine CNT supports and those after 24 hours of immersion in various solvents. (d) I_D_/I_G_ ratio of each sample in (c).


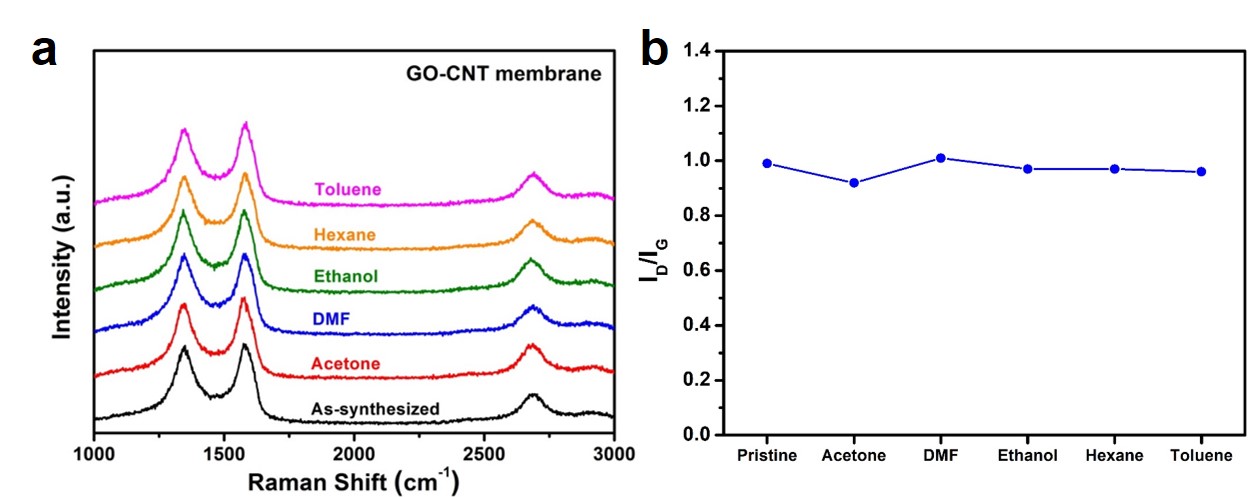


Supplementary Figure S10. Chemical stability of GO-CNT membranes. (a) Raman spectra of pristine GO-CNT membranes and those after 24 hours of immersion in various organic solvents. (b) I_D_/I_G_ ratio of each sample in (a).


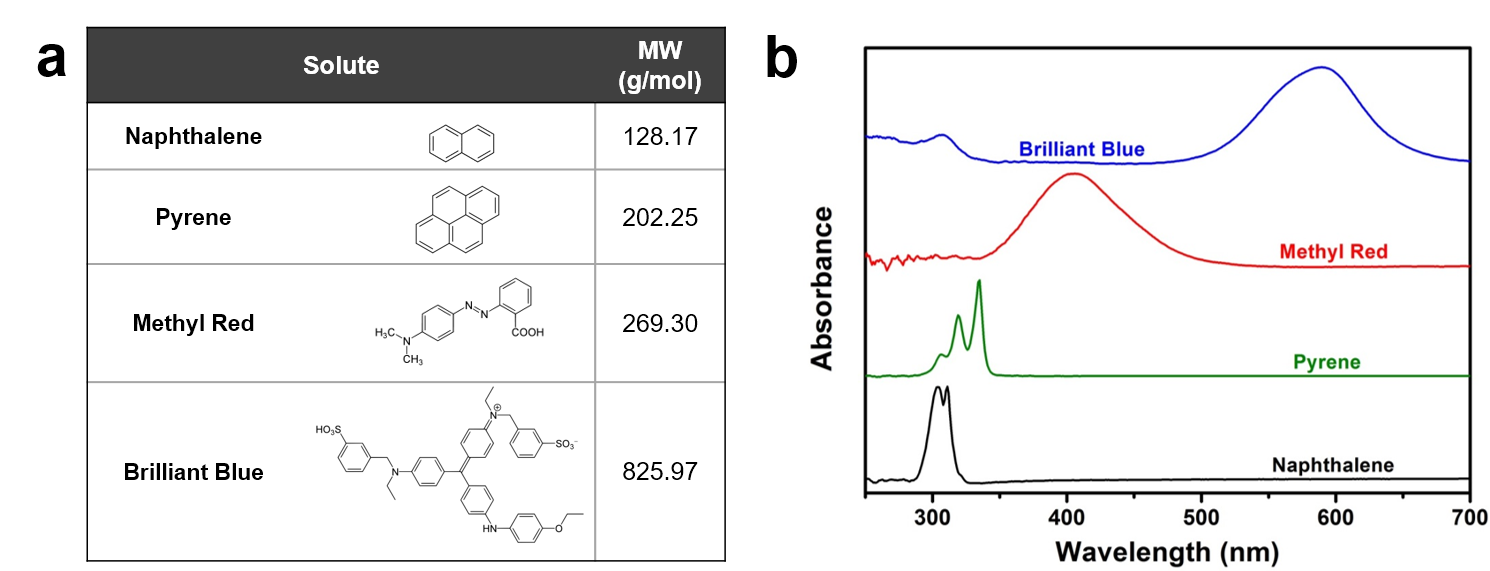


Supplementary Figure S11. Solutes employed for permeation tests. (a) Molecule configuration, and molecular weight (MW) for four different solutes: naphthalene, pyrene, Methyl Red, and Brilliant Blue. (b) UV spectra of typical solutions of various solutes in ethanol.


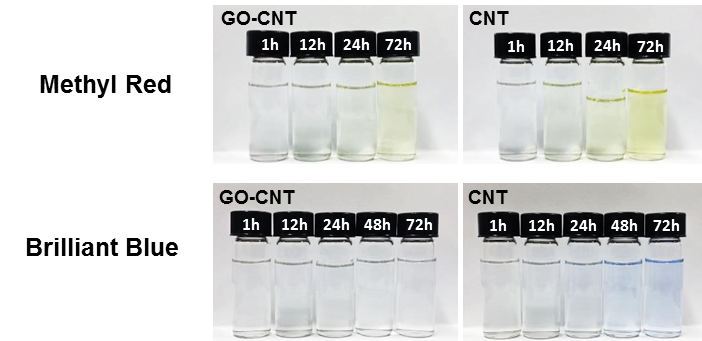


Supplementary Figure S12. Images of permeated solutions through membranes according to time. Images on the top represent Methyl Red in ethanol permeated through GO-CNT and CNT membranes, and images on the bottom represent Brilliant Blue in ethanol permeated through the same. Selective permeation of Brilliant Blue through GO-CNT can be directly observed.


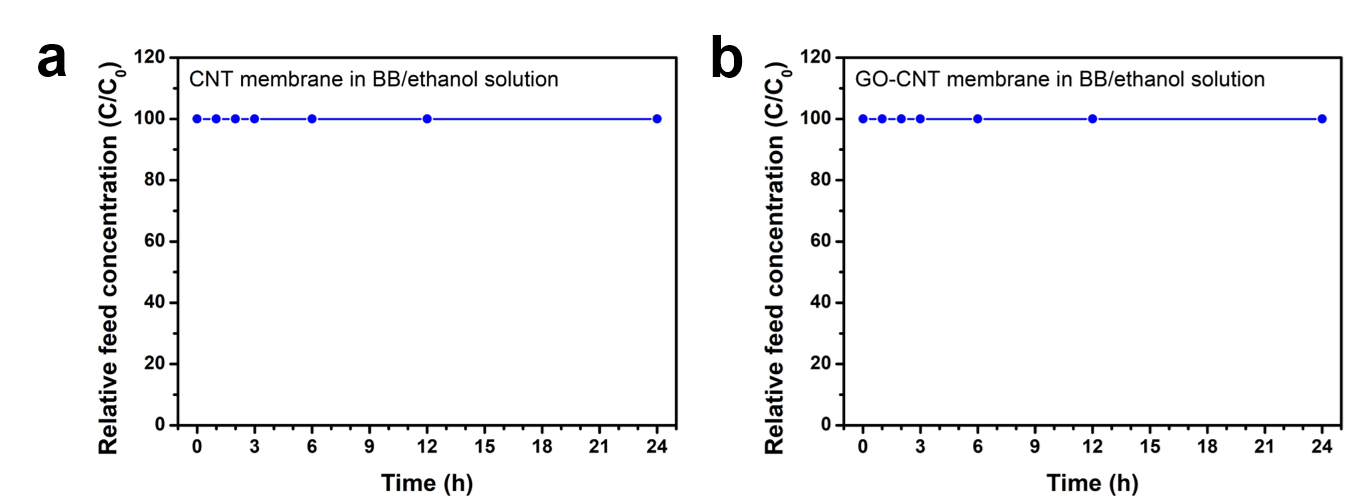


Supplementary Figure S13. Influence of dye adsorption on feed concentration. (a, b) Relative feed concentration (C/C_0_) after immersing (a) CNT membrane, and (b) GO-CNT membrane in 10 mg/L BB/ethanol solutions up to 24 hours. Results indicate that the concentration change from dye adsorption on membranes is negligible.


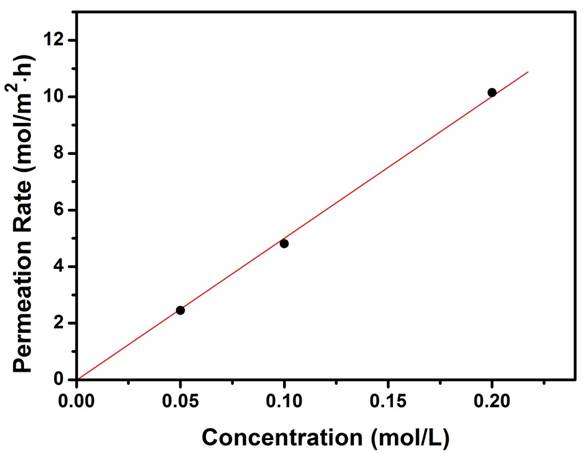


Supplementary Figure S14. Permeation rate of naphthalene in ethanol in various concentrations through GO-CNT membranes. Rates were measured at three different concentrations, which display a linear relationship.


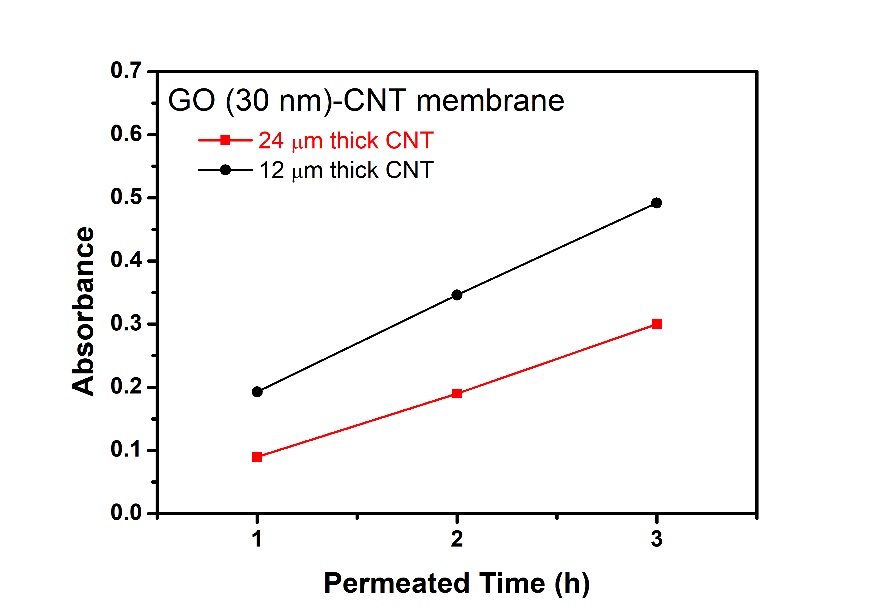


**Supplementary Figure S15.** Permeation rate of naphthalene in ethanol through GO (30 nm)-CNT membranes, according to CNT thickness
